# Formation Mechanism of Well-Ordered Densely Packed Nanoparticle Superlattices Deposited from Gas Phase on Template-Free Surfaces

**DOI:** 10.1186/s11671-021-03635-7

**Published:** 2021-11-30

**Authors:** Chang Liu, Fei Liu, Chen Jin, Sishi Zhang, Lianhua Zhang, Min Han

**Affiliations:** 1 National Laboratory of Solid State Microstructures and Collaborative Innovation Centre of Advanced Microstructures, Nanjing University, Nanjing, 210093 China; 2 Department of Materials Science and Engineering and Jiangsu Key Laboratory of Artificial Functional Materials, Nanjing University, Nanjing, 210093 China

**Keywords:** Ordered nanocluster monolayer, Gas-phase cluster deposition, Self-assembling, Densely packed, Surface migration, Attractive interaction

## Abstract

Superlattices of nanoparticles are generally produced based on solution chemistry processes. In this paper, we demonstrate that self-assembled monolayer structures of nanoparticles with superlattice periodicities can also be produced on template-free surfaces in the gas-phase cluster beam deposition process. It is found that the packing of Fe nanoparticles corresponds to an average of two-dimensional densely packed lattice with a hexagonal summary. By controlling the nanoparticle coverage, the two-dimensional densely packed monolayer morphology can spread to the whole substrate surface being deposited. A formation mechanism of the ordered monolayers is proposed. The densely packed morphologies are formed by the balance between the diffusion rate of the nanoparticles and their filling speed on the substrate surface determined by the deposition rate, and the ordering of the nanoparticle arrays is driven by the inter-particle attractive interactions. The model is strongly supported by a series of carefully designed cluster deposition experiments.

**Supplementary Information:**

The online version contains supplementary material available at 10.1186/s11671-021-03635-7.

## Introduction

Nanoparticle superlattices, in which the particles behave as artificial atoms and are arranged with crystallographic orders, have attracted tremendous attention since they were first reported [1–7]. Superstructures assembled from tailored nanoparticle/nanocrystal building blocks enable the design of novel materials and optimizing and/or tuning the properties and performance of the nanomaterials [8–16]. As an elegant alternative to the lithographic approaches, nonlithographic bottom-up approaches based on thermodynamically driven self-organization processes are especially appealing, because of their advantages such as simpler technology and potential for large-scale production of very small structures at scales beyond the current limits of lithographic techniques.

Generally, ordered arrays or superlattices of nanoparticles are produced based on solution chemistry processes. Nanoparticles/nanocrystals from a colloidal solution can form ordered arrays upon the spontaneous organization of monodispersed nanoparticles/nanocrystals encapsulated in surfactant monolayers [17–20], or by using biological molecules and their specific interactions [21, 22]. Typically, the nanostructure assembled from colloidal solution has organic monolayers to encapsulate the particles, which produces a soft structure. The soft ligands can prevent nanoparticles from disordered aggregation, fine-tune the interparticle potential as well as program lattice structures and interparticle distances [23, 24]. On the other hand, such chemical additive often facilitates tailoring the intrinsic property of the nanoparticle assembly and sometimes becomes a significant limitation [25].

In recent years, efforts were also devoted to fabricating nanoparticle arrays with predeterminate patterns from gas phase. Unlike the nanostructures from a colloidal solution, nanoparticle assemblies formed in the gas phase do not have organic surface encapsulates, so that pure inter-particle interfaces and intrinsic properties can be expected. However, to fabricate nanoparticle assemblies with controlled spatial organizations, various templates with pre-patterned surface features have to be used. By performing gas-phase atom deposition, ordered arrays of uniform nanoparticles were fabricated via strain-induced spontaneous nucleation of three-dimensional nanometer-sized islands on top of a strained epilayer [26, 27]. Arrays of quasi-one-dimensional Ag nanoparticle chains were generated by trapping the gas-phase deposited nanoparticles at the step edges of graphite surface [28]. Shi et al. used self-assembled nano-patterns of block copolymer as templates for gas-phase cluster deposition to produce two-dimensional (2D) arrays of metal nanoparticles [29]. Although selective decoration of phase-separated diblock copolymer template [30] with vacuum-deposited metals has become a common way to fabricate patterned nanoparticle arrays, the assembly such produced contained many defects and the packing of the nanoparticles was not so compact due to the relatively large spatial period in the self-assembled patterns of the block copolymer templates. Sintering or post-processing the nanoparticles on the substrate is another way to obtain nanoparticle aggregations, but only irregular products can be obtained [31]. Up to now, it is unsuccessful to assemble ordered arrays of nanoparticles via gas-phase deposition on template-free surfaces.

Gas-phase nanocluster deposition provides a well-developed process able to produce nanoparticle-based nanostructures with a high level of control on size, density, and functional assembling morphology [32–35]. Generally, nanoparticles deposited at low kinetic energy are able to diffuse on the surface with high mobility and tend to aggregate [36]. On the other hand, the migrations of the nanoparticles are constrained by surface defects. Therefore, in previous studies, either random arrays [37] or large ramified aggregates [38] of nanoparticles were obtained with a moderate deposition mass, depending on the impact energy of the nanoparticles and the substrate. It was commonly believed that such structures are strongly disordered with neither long-range orders nor short-range orders.

In this paper, we demonstrate that well-ordered densely packed nanoparticle monolayers could be formed on the smooth surface of amorphous carbon substrate by performing gas-phase cluster deposition without any preformed template. With moderate coverage, ordered arrays of densely packed nanoparticles are observed in the submicron length scale. The nanoparticle monolayers display a certain superlattice periodicity. We carry out a series of cluster deposition experiments to analyze the formation mechanism of the nanoparticle superlattice morphology, by considering a balance among various factors of cluster deposition dynamics, such as the flux and the kinetic energy of the clusters, as well as the migration ability of clusters on the substrate surface. This finding provides an effective route to fabricate 2D nanoparticle superlattice structures by gas-phase deposition process, which can be an alternative to the solution chemistry-based method, with the advantages such as rapid and simple procedures, clean surfaces and interfaces, as well as high stabilities.

## Methods

### Deposition of Nanoparticle Arrays

**Fig. 1:**
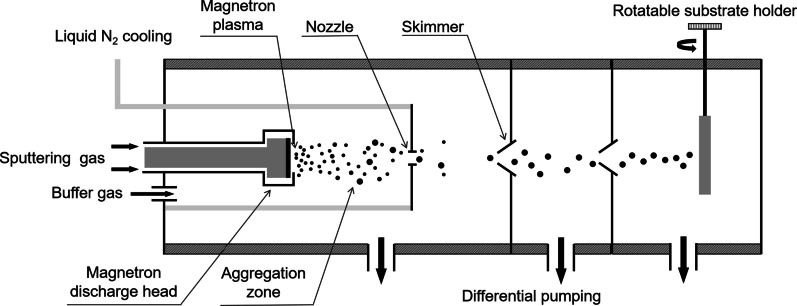
Schematic diagram of a magnetron plasma gas aggregation cluster source and the cluster beam deposition process

Fe and TiN nanoparticles were generated by using a magnetron plasma gas aggregation cluster source [37, 39] consisting of a liquid nitrogen cooled aggregation tube ended with an orifice 3 mm in diameter. A schematic drawing depicting this setup is presented in Fig. 1. The magnetron discharge was operated at a pressure of 64 Pa in argon stream. Atoms were sputtered from the target on the magnetron discharge head and aggregated into clusters in the argon stream. An aggregation length (the length of the space where cluster formation took place) of 75 mm was used. The clusters were swept by the argon gas stream out of the aggregation tube into a high vacuum (< 1 × 10^4^ Pa) chamber through the orifice and formed a collimated nanoparticle beam with a divergence of about 3°. The nanoparticles were deposited on the substrates fixed on a rotary sample holder in the high vacuum chamber. The incident angle of the nanoparticles on the substrate could be tuned between 0° and 90° by rotating the sample holder. The deposition rate was monitored with a quartz crystal microbalance and controlled precisely by the discharge power applied to the cathode with a DC power supply (MDX500, Advanced Energy). During the deposition, the substrates are kept at room temperature.

### Characterizations of the Nanoparticle Arrays

The nanoparticles were deposited on ultrathin amorphous carbon films on formvar-coated 300-mesh copper grids. The structure and morphology of the deposited nanoparticle films were characterized with a transmission electron microscope (TEM, FEI TECNAI F20s TWIN) operated at 200 kV. The composition of the nanoparticles was analyzed using energy-dispersive X-ray spectroscopy (EDX). To analyze the oxidation state of the Fe nanoparticle arrays, X-ray photoelectron spectroscopy (XPS) was carried out with an ESCALABMK-II spectrometer using a monochromatic MgKα source.

Fe nanoparticles were also deposited on silicon wafers for magnetization measurements, which were performed by using a superconducting quantum interference device magnetometer (SQUID, MPMS-3).

## Results and Discussion

### Analysis of the Structure of the Nanoparticle Film

**Fig. 2:**
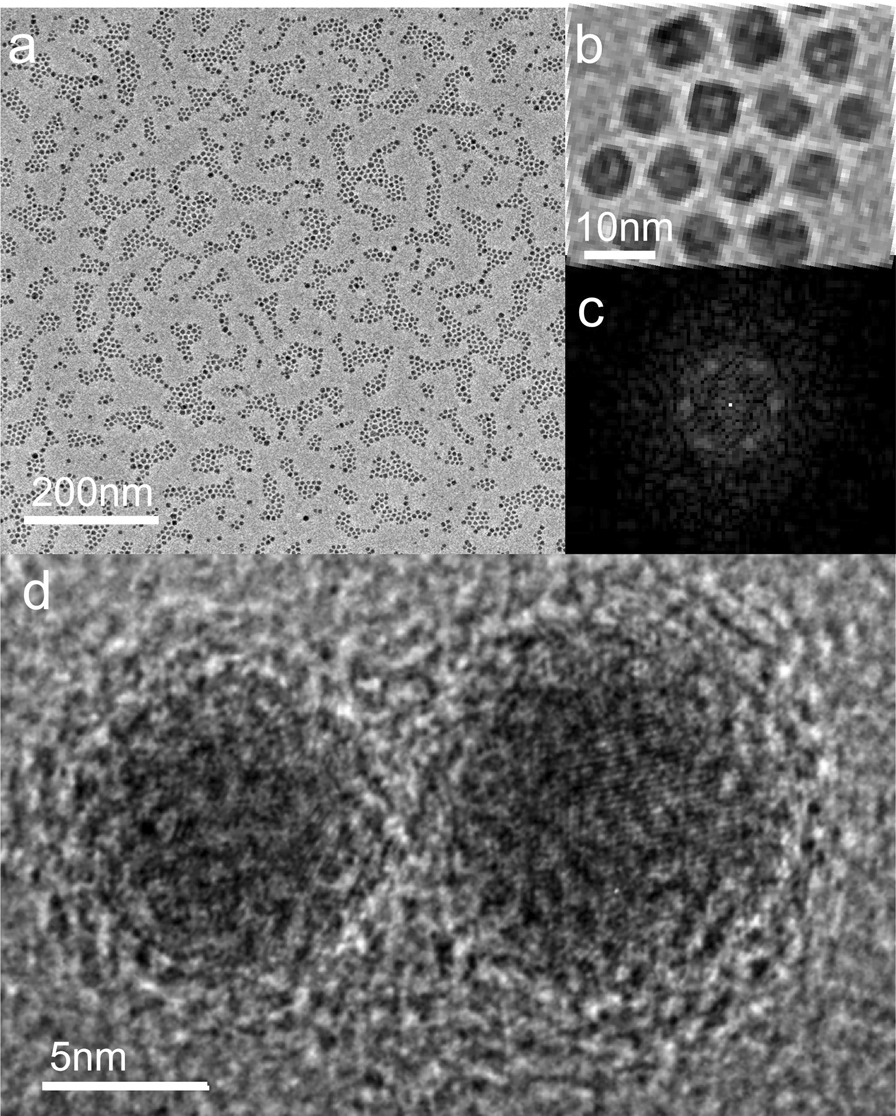
**a** Low-magnification TEM image of Fe nanoparticle arrays deposited on a TEM grid covered by amorphous carbon film, with a deposition rate of 0.1 Å s^−1^. Domains of close-packed 2D monolayers with long orders can be identified. **b** High-magnification TEM image of an ordered densely packed 2D superlattice of Fe nanoparticles. **c** FFT corresponding to nanoparticle arrays in (**a**). **d** HTREM image shows lattice images of individual Fe nanoparticles

Figure 2a shows the TEM image of Fe nanoparticle arrays prepared by depositing the nanoparticle beam vertically on the surface of the amorphous carbon film with a deposition rate of 0.1 Å s^−1^. The deposition time is 5 min. The nanoparticle array is composed of densely packed 2D monolayer domains ordered over 100–200 nm scale. A high-magnification TEM image of a well-ordered defect-free monolayer domain is shown in Fig. 2b. Typically, the nanoparticle monolayers may contain some defects, such as lattice distortions, dislocations, vacancies, or voids, as well as size variations of the nanoparticles. The average nanoparticle size is 6.1 ± 1.6 nm, as determined using a minimum of 300 nanoparticles in the arrays (Additional file 1: Fig. S1). The size dispersion is significantly larger than those in the self-assembled superlattices of thiol-passivated nanoparticles [40, 41]. A high-resolution transmission electron microscopy (HRTEM) image given in Fig. 2d shows that the individual Fe nanoparticles are mainly single crystals with spherical shapes. They are randomly oriented on the substrate surface. Prior to the observation, the nanoparticles have been exposed to air for a significantly long time so that their surfaces are sufficiently oxidized, as can be distinguished in the HRTEM image. The existence of the oxidation layer on the nanoparticle surface can be further confirmed by EDX and XPS. As shown in Additional file 1: Fig. S2, O elements are always observed together with the Fe nanoparticles in the EDX elemental mapping images. XPS measurements also show the evidence of oxidation of Fe nanoparticles. As shown in Additional file 1: Fig. S3, both metallic Fe and Fe oxides can be distinguished from the photoemission data of the Fe 2*p* core levels. After the nanoparticle specimens are cleaned with Ar ion sputtering, the XPS peaks corresponding to the 2*p* core levels of pure Fe are greatly enhanced, indicating that Fe oxides are only present on the nanoparticle surfaces. Therefore, the nanoparticle arrays can be considered as a compact packing of closely contacted core/shell nanoparticles. The crystalline metal cores are separated from each other with amorphous oxide shells. The oxide shell acts as a passivation layer preventing further oxidation of the Fe nanoparticles. The mean edge-to-edge distance separating well-aligned nanoparticles in the closely packed lattice is measured at 1.7 ± 0.6 nm. Correspondingly, the thickness of the amorphous oxide shell is about 0.85 nm on average. It should be noted that the oxide shells are formed after the deposition process is finished. Since the nanoparticle deposition is performed under a high vacuum condition, the self-assembling occurs in the pure metal nanoparticles, rather than the surface-oxidized nanoparticles. The amorphous oxide shells play no role in the organization of the nanoparticles.

The fast Fourier transform (FFT) of the densely packed monolayers is shown in Fig. 2c. Well-defined spots arranged in hexagon are shown, attesting to a densely packed nanoparticle lattice ordered over a long range. However, only one hexagon related to the first order is distinguishable, indicating that the scale of the ordered monolayer domains is limited.

### Tailoring the Assembling Morphology with Nanoparticle Deposition Conditions

**Fig. 3:**
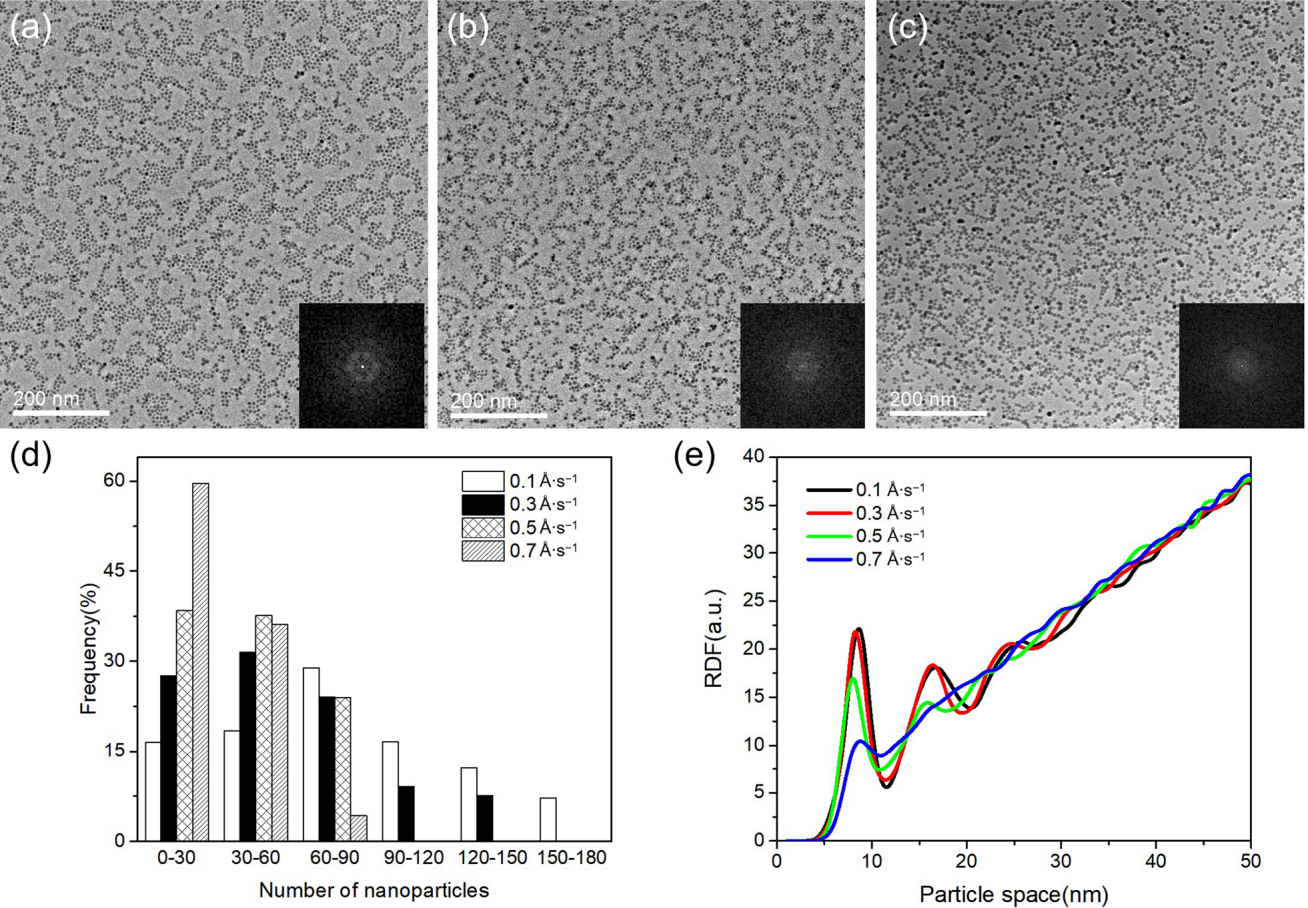
TEM images of Fe nanoparticle arrays with different deposition rates: **a** 0.3 Å s^−1^, **b** 0.5 Å s^−1^, **c** 0.7 Å s^−1^. The insets show the fast Fourier transform of each spatial distribution. **d** Histograms of nanoparticle numbers contained within an individual monolayer domain in the nanoparticle arrays deposited with different deposition rates. The average nanoparticle numbers within an individual monolayer domain are counted to 77, 55, 39, and 27 for deposition rates of 0.1 Å s^−1^, 0.3 Å s^−1^, 0.5 Å s^−1^, and 0.7 Å s^−1^, respectively. **e** Radial distribution function of the Fe nanoparticle arrays with different deposition rates

We have found that the deposition rate of the nanoparticles plays a definite role in the formation of the ordered densely packed monolayers. In Fig. 3a–c, TEM images of Fe nanoparticle arrays prepared with deposition rates ranging from 0.3 to 0.7 Å s^−1^ are shown. The FFT of each image is shown as the insets. The deposition time of each specimen is such controlled that a constant nanoparticle coverage (i.e., total deposition mass) on the substrate is maintained. In each image, the average nanoparticle size and distribution (Additional file 1: Fig. S1) are almost identical. (The average diameter is measured to be 6.0 ± 1.4 nm, 6.1 ± 1.3 nm, and 6.1 ± 1.7 nm, respectively). From Figs. 2a and 3a–c, we can find that with the increase in the deposition rate, the range scale of the ordered monolayer domains becomes smaller and smaller. We analyze the TEM images by counting the nanoparticle numbers contained in each monolayer domain. The sizes of the monolayer domains can be compared quantitatively with the nanoparticle numbers they contain. The histograms of the counted nanoparticle numbers are shown in Fig. 3d. To keep a reasonable statistic, a dozen of TEM images is analyzed for each deposition rate. The maximum of the distribution tends to smaller nanoparticle numbers with the increase in deposition rate. The average nanoparticle number contained in an individual monolayer domain decreases from 77 at a deposition rate of 0.1 Å s^−1^ to 27 at a deposition rate of 0.7 Å s^−1^. Meanwhile, the spots present in the FFT pattern become more and more diffuse. With a deposition rate of 0.7 Å s^−1^, only a diffuse ring without any hexagonal symmetry can be seen in the FFT pattern. The nanoparticles in the TEM image display a random distribution on the whole.

In Fig. 3e, the radial distribution functions (RDFs) calculated from the TEM images are shown. For the nanoparticle arrays formed at 0.1 Å s^−1^ and 0.3 Å s^−1^ deposition rates, the RDF curves display sharp and clear first and second peaks, corresponding to the nearest- and second-nearest neighbors with average particle–particle intervals of 8 nm and 17 nm, and a distinguishable third peak corresponding to the third neighbors with an average interval of 24 nm, indicating that the nanoparticle arrays are well-ordered with superlattice periodicities. For the nanoparticle arrays formed at 0.5 Å s^−1^ deposition rate, the second peak in the RDF curve becomes much reduced and the third peak is completely indistinguishable, indicating a decreasing organization and reduced lattice periodicity. With a deposition rate of 0.7 Å s^−1^, the nanoparticle arrays only display a weak first peak in the RDF curve, which strongly reflects the loss of the lattice periodicity and short-range order. It is clear that a low deposition rate is an important parameter dominating the well-ordered monolayer formation.

**Fig. 4:**
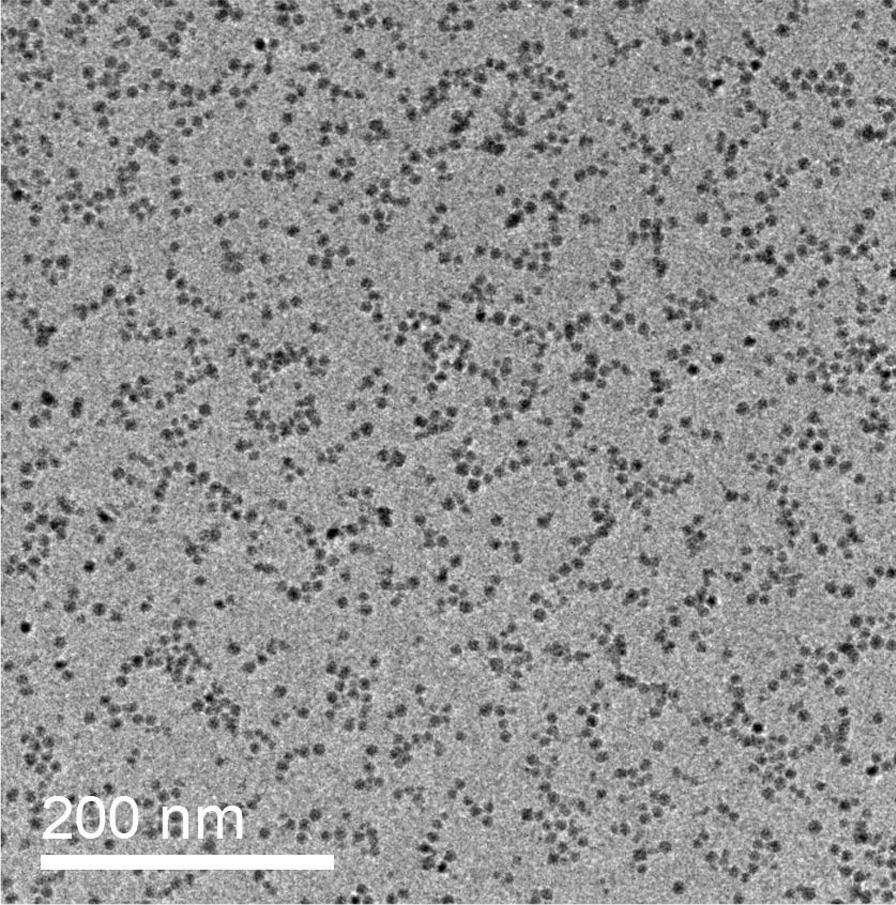
TEM image of Fe nanoparticle arrays deposited on a Formvar film with a deposition rate of 0.1Å∙s^−1^

We have also found the structure of the nanoparticle arrays is correlated with the feature of the substrate surface. Different assembling patterns are obtained with different substrates. Figure 4 shows a TEM image of the Fe nanoparticle arrays deposited on a Formvar film. The deposition is performed with a deposition rate of 0.1 Å s^−1^. Although the operation parameter of the cluster source and the deposition mass is identical to that used for the sample shown in Fig. 2, ordered densely packed morphology could no more be observed. The distribution of the nanoparticles on the surface is completely random. No evidence of organization could be observed. In some areas, homogeneous coalescences of the nanoparticles form larger particles. It is known that the mobility of nanoparticles softly landing on solid surfaces is strongly dependent on the nature of the surface [33], especially its defect state and binding ability with the deposits. It has been shown that metal nanoparticles have high mobilities on the surface of carbon materials [36, 37]. It was demonstrated that metal nanoparticles deposited at low energy can diffuse across the graphite surface freely and tend to aggregate. On highly oriented pyrolytic graphite (HOPG), which has an atomically smooth surface, metal nanoparticles are able to diffuse with high mobility and be trapped by surface defects. At low coverage, most of the deposited nanoparticles decorate step edges and point defects on the terraces. At higher coverage, the diffusion and aggregation of the nanoparticles on the carbon surface lead to large ramified island structures or random packing morphologies, depending on the density of the defects [42]. It was also observed that diffusion and coalescence of metal nanoparticles on amorphous carbon surfaces can induce particle size gradient from the gradient of nanoparticle coverage [39]. On the contrary, no diffusive aggregation of metal nanoparticles has been observed on the Formvar film surface [43]. Although the number density of the nanoparticles increases significantly, they mostly keep isolated from each other and little coagulation among adjacent particles can be observed. Instead, the nanoparticles are mostly pinned where they are deposited. It is difficult for them to diffuse and aggregate on the substrate. Coalescence takes place locally as a fusion process under particle–particle collision in the deposition process. These results suggest that certain mobility is needed once the nanoparticles are deposited on the surface in order to form densely packed monolayers.

To understand the formation mechanism of the densely packed nanoparticle monolayers in the gas-phase cluster deposition, we have to consider the competition between the diffusion rate of the nanoparticles and the filling speed of the nanoparticles deposited on the substrate surface, which is dependent on the deposition rate. This is similar to the situation that happens in the spontaneous organization process occurring at the liquid/substrate interface to form periodic 2D arrays of thiolate-encapsulated nanoparticles upon solvent evaporation from a droplet of colloidal solution depositing on the substrate. Previously, experiments [44, 45] showed that when a droplet of nanoparticle solution was deposited onto a substrate and dried shortly, amorphous nanoparticle aggregates with little uniformity and symmetry were formed. As the droplet was dried more and more slowly, increasing uniformity was observed and finally closely packed nanoparticle superlattices were formed. With a slow solvent evaporation rate, the nanoparticles benefit from more time to diffuse on the substrate and adjust their sites attached to the nanoparticle assembly, giving rise to a higher level of ordering. Similarly, the nanoparticles deposited on the carbon substrate from gas phase can diffuse on the free surface with high mobility. If the arrival rate of nanoparticles to the surface is too high, the motion of the nanoparticles on the surface will be limited by each other, and the free area available for each nanoparticle will be soon exhausted. The nanoparticles cannot sufficiently adjust their positions on the surface, resulting in randomly packed aggregates. Moreover, if the sticking coefficient between the nanoparticles remains high, low-density fractal aggregates are formed [36, 38]. However, with a mild deposition rate, the arrival time of the nanoparticles is controlled such that the nanoparticles have enough time to diffuse on the free surface and find equilibrium lattice sites on the growing structure. As a result, ordered densely packed monolayers are formed. As the flux of nanoparticles adding to the surface is increased by increasing the deposition rate, the arrival rate exceeds the surface mobility of nanoparticles and the formation of an inhomogeneous disordered aggregate occurs.

**Fig. 5:**
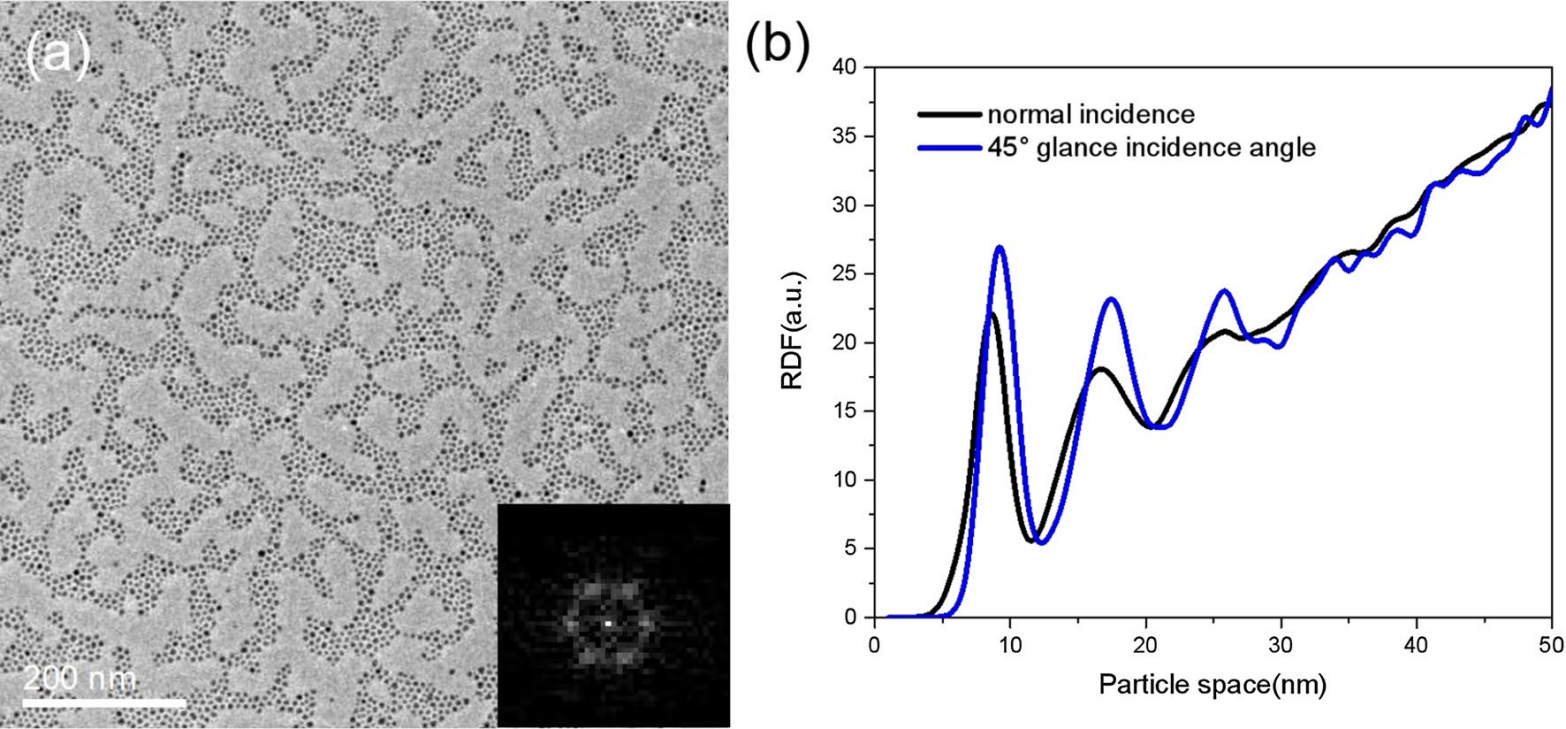
**a** TEM image of Fe nanoparticle arrays deposited with a 45° glance incidence angle. The inset shows an FFT of the image. **b** A comparison of the RDFs of the Fe nanoparticle arrays deposited with a normal incidence and a 45° glance incidence

The surface mobility of the nanoparticles is dependent on the interaction between the nanoparticle and the surface. On the surface of organic materials, metal nanoparticles are mostly pinned where they are deposited. Although they have high mobility on the perfect surface of carbon substrate, their diffusion may also be limited by the particle diffusion barriers on the surface, such as the defects. If the thermal energy of a nanoparticle is low compared with the binding energy, it may be arrested on the diffusion barriers. It is possible to increase the diffusion length of the nanoparticles by increasing their lateral migration energies when they land on the surface. We try to increase the lateral migration energies of the nanoparticles by increasing their momentums along the surface when they impact the substrate. This is achieved by depositing nanoparticles with a glance incidence relative to the substrate surface. The lateral migration energies of the nanoparticles increase due to the increasing of their momentums along the surface when they impact on the substrate. Generally, the initial kinetic energy of the nanoparticles generated from a cluster source is several eV on average. With a glance incidence, a partial of the kinetic energy transfers to the migration energy of the nanoparticle on the surface. This will enhance the abilities of the nanoparticles to escape from the diffusion barriers where they are arrested, so as to increase the migration length of the nanoparticles. In Fig. 5a, a TEM image of the Fe nanoparticle arrays prepared with a 45° glance incidence angle is shown. The equivalent deposition rate is 0.1 Å s^−1^. Comparing with the nanoparticle arrays prepared with the same deposition parameters under normal incidence (Fig. 2a), we find the range scale of the ordered monolayer domains is significantly increased, and the hexagonally arranged FFT spots become sharper, clearer, and more scattered. From the RDF curve shown in Fig. 5b, we can see the second-nearest peak is notably enhanced and sharpened. Especially, the third neighbor peak, which is indistinct in the case of the normally deposited samples, becomes sharp and clear now, indicating a significant improvement in the organization length and lattice periodicity. It, therefore, demonstrates a simple way to increase the diffusion length of the nanoparticles so as to realize larger-scale ordered monolayers.

**Fig. 6:**
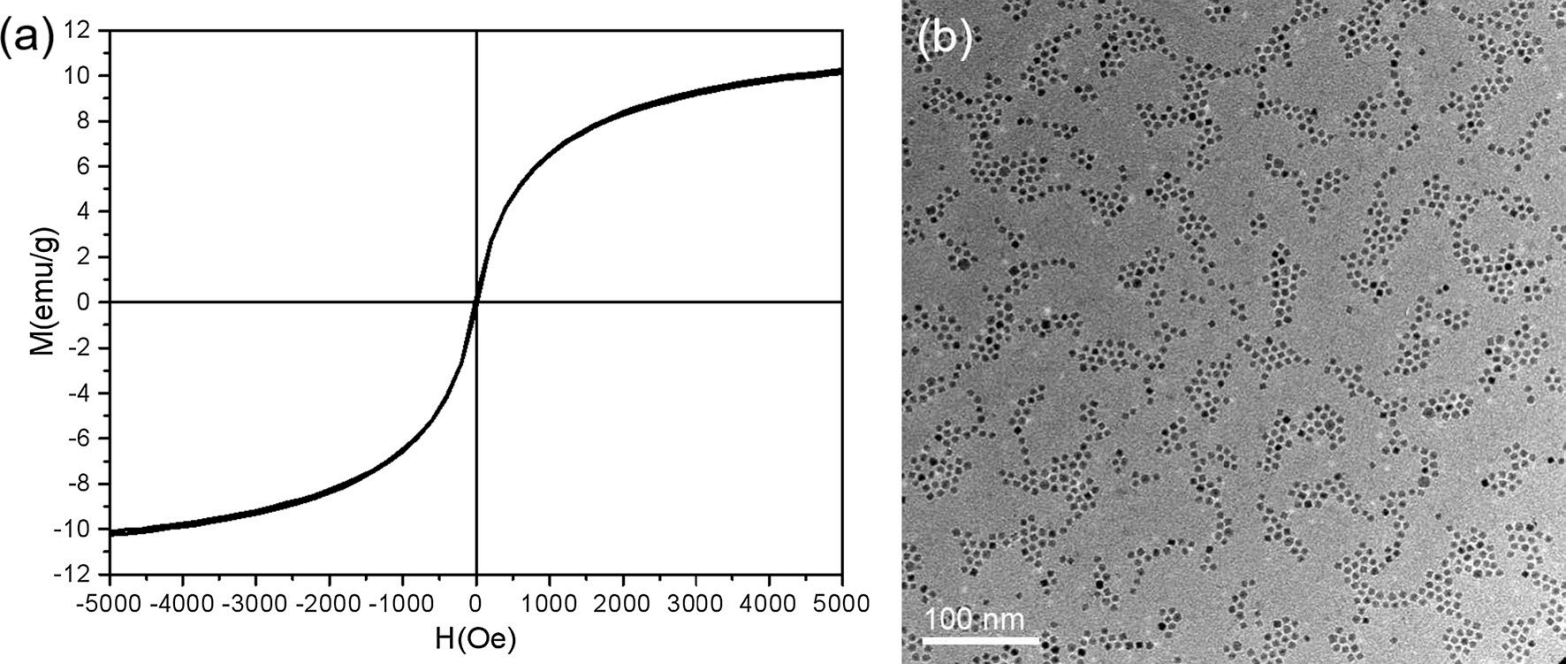
**a** Hysteresis loop measured at 300 K of densely packed 2D self-assembled Fe nanoparticle arrays, showing that the nanoparticles are in the superparamagnetic states. **b** TEM image of TiN nanoparticle arrays deposited on a TEM grid covered by amorphous carbon film, showing that the nanoparticle arrays are composed of domains of ordered densely packed 2D monolayers

It should be noted that the balance between the diffusion rate and the arrival time of the nanoparticles on the surface is not the only condition sufficient for the ordered nanoparticle monolayer formation. The ordering is driven by the interparticle attractive forces. Unlike in the case of self-assembled superlattices of thiolate-encapsulated nanoparticles, in which the main contribution to the interaction comes from the surfactant molecules, which produce a soft structure [41], in the present study, it is the attractive van der Waals force between neighboring nanoparticles that dominate the ordered nanoparticle array formation, which produces a rigid hard structure (Additional file 1: Note 1). In the 2D densely packed monolayer, a nanoparticle falls on the equilibrium site since it receives the maximum attractive interactions from the identical nearest neighbors. With a sufficiently long time for free diffusion, the individual nanoparticles can sufficiently modify their positions to find the equilibrium lattice positions. A challenge comes from that Fe is a ferromagnetic material. The dipolar magnetic interactions between magnetic nanoparticles increase with the particle volumes and oppose any 2D long-range ordering. Previous studies showed that Co nanoparticles larger than 16 nm tended to form one-dimensional chains and a variety of linear structures [46]. Therefore, in the present case, magnetic interactions play no role in the self-assembling of the 2D densely packed monolayers of Fe nanoparticles. In fact, magnetization measurements on the Fe nanoparticle deposits display no ferromagnetic hysteresis loops and remnant magnetizations around room temperature, as shown in Fig. 6a, indicating that the Fe nanoparticles are in the superparamagnetic states. It is more likely that attractive van der Waals interactions or dipolar interactions arisen from polarizations dominate the self-assembling of the ordered 2D densely packed monolayers. In fact, we can also obtain ordered densely packed monolayers from nanoparticles of nonmagnetic materials. TiN nanoparticles are generated in the gas aggregation cluster source and deposited on the amorphous carbon with similar deposition conditions. From the TEM image shown in Fig. 6b, we can see most of the TiN nanoparticles are involved in a number of ordered monolayers with 2D densely packing superlattice structures. Similar to the case of Fe nanoparticles, the TiN nanoparticle superlattices can spread over hundreds nm scales.

Based on the above discussion, the formation process of nanoparticle superlattice in the gas-phase nanoparticle deposition can be summarized as follows: At the very beginning of deposition, nanoparticles are preferentially trapped on surface defects during their migrations on the substrate surface. They play as ‘nuclei’ and random aggregates develop by the adding of subsequently deposited nanoparticles during their migration on the substrate surface. After the nanoparticles add to the aggregates, their kinetic energies can still enable them to modify their positions locally in the aggregates and find equilibrium lattice sites on the growing structures. The nanoparticle falls on the equilibrium site since a local minimum of interaction energy between the nanoparticle and the identical nearest neighbors is reached. As a result, 2D densely packing nanoparticle superlattice structures form. However, the kinetic energies of nanoparticles are not sufficient to enable them to jump out the local minimum so as to explore the global minimum. In this case, ordered nanoparticle arrays with irregular shapes are commonly observed.

**Fig. 7:**
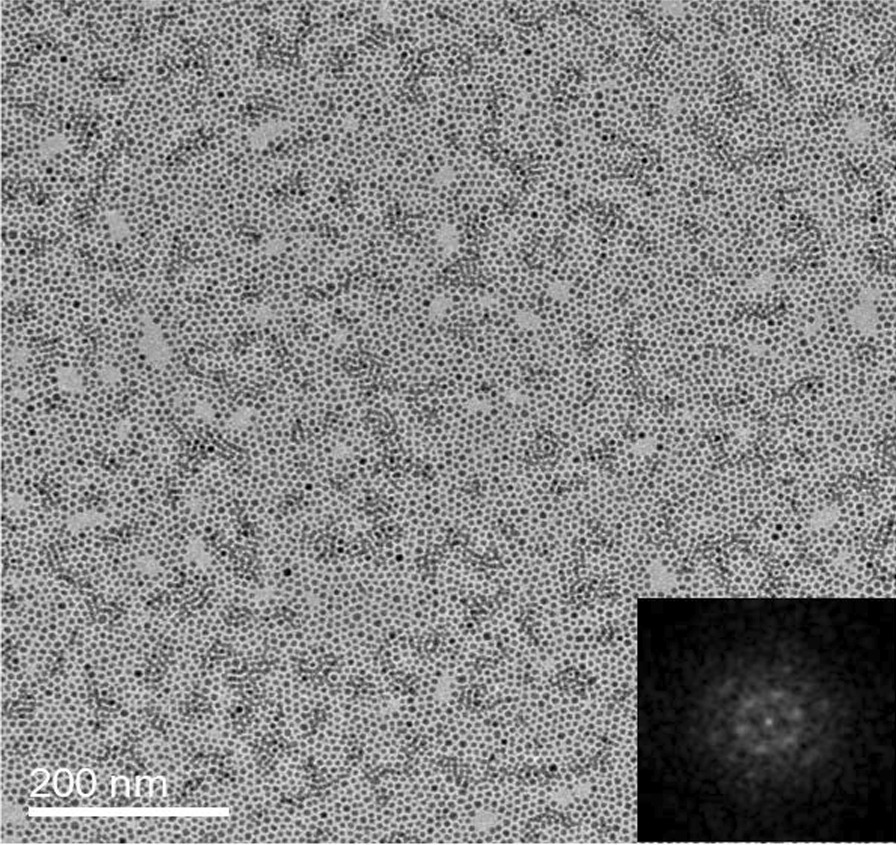
TEM image of Fe nanoparticle film with nanoparticle coverage approaching 100%. The deposition is performed at an equivalent deposition rate of 0.1 Å s^−1^ with a 45° glance incidence. The boundaries between the ordered monolayer domains are hardly identified. The inset shows the FFT of the nanoparticle assembling structures

Regarding the size of the ordered densely packed 2D monolayer structures that can be achieved with the gas-phase cluster deposition, we show in Fig. 7 a TEM image of Fe nanoparticle film with a coverage approaching 100% (i.e., a complete monolayer). By controlling the deposition mass, the densely packed 2D monolayer structure spreads over the whole substrate surface covered by the deposition spot (at least at the centimeter scale). The monodispersed nanoparticles show a perfect homogeneous distribution in the wide range. Only a few several tens of nanometer-sized voids distribute in a very low density. The FFT of the monolayer (inset in Fig. 7) shows two rings of hexagonally arranged spots, related to the first and second orders, attesting to a well-defined hexagonal network ordered over a sufficiently long range. Even though the large-scale assembling structure contains domains of ~ 100 nm in size, with a number of packing arrays or orientations of the same structure, it is difficult to find any well-defined boundaries between the ordered domains. This result demonstrates the gas-phase cluster deposition may provide an efficient way for the fabrication of well-defined patterned superstructures assembled from nanoparticle building blocks on a sufficiently large scale.

## Conclusion

We have demonstrated a simple, fast, and convenient approach to the fabrication of ordered densely packed 2D self-assembled monolayer structures of Fe nanoparticles on the template-free surface such as amorphous carbon film by performing gas-phase cluster deposition with finely controlled deposition rate and deposition time. This approach has allowed us to prepare 2D superlattice domains composed of well-defined hexagonal nanoparticles networks ordered over lateral dimensions of 100–200 nm. We have carried out a series of cluster deposition experiments by carefully varying the nanoparticle deposition dynamics, such as the flux, the lateral migration energy, as well as the migration ability of nanoparticles on the surface. The experimental evidence indicates that the 2D self-assembled monolayer structures are formed by the balance between the diffusion rate of the nanoparticles and their filling speed on the surface, which is dependent on the deposition rate. Meanwhile, the attractive interactions between the nanoparticles drive the ordering in the densely packed arrays. Such a mechanism has also allowed us to deposit ordered densely packed self-assembled monolayer structures of nonmagnetic materials, such as TiN. By controlling the total deposition mass, the densely packed 2D monolayer domains can spread homogeneously over the whole substrate surface covered by the deposition spot. We believe that this method will provide an alternative to the solution chemistry-based method that has been commonly used for the fabrication of periodic 2D arrays of thiolate-encapsulated nanoparticles from spontaneous organization.

## Supplementary Information

**Additional file 1.** The following files are available free of charge. Size distribution of Fe nanoparticles; the element distribution of Fe nanoparticle arrays; oxidation state of Fe nanoparticle arrays; heavily deposited Fe nanoparticle film; Fe nanoparticle arrays on the surface with different defect densities; surface morphology of amorphous carbon films; Fe nanoparticle aggregates deposited under nonoptimum condition; **Note 1.** On the rigid hard structure formed with metal nanoparticles without surfactant; **Note 2.** On the effects of surface texture of the amorphous carbon substrate.

## Data Availability

The datasets used and/or analyzed during the current study are available from the corresponding author on reasonable request.

## Abbreviations

TEM: Transmission electron microscope
EDX: Energy-dispersive X-ray spectroscopy
XPS: X-ray photoelectron spectroscopy
HRTEM: High-resolution transmission electron microscopy
FFT: Fast Fourier transform
RDFs: Radial distribution functions

## References

[1] Si KJ, Chen Y, Shi Q, Cheng W (2018). Nanoparticle superlattices: the roles of soft ligands. Adv Sci.

[2] Boles MA, Engel M, Talapin DV (2016). Self-assembly of colloidal nanocrystals: from intricate structures to functional materials. Chem Rev.

[3] Boneschanscher MP, Evers WH, Geuchies JJ, Altantzis T, Goris B, Rabouw FT, van Rossum SA, van der Zant HS, Siebbeles LD, Tendeloo GV, Swart I, Hilhorst J, Petukhov AV, Bals S, Vanmaekelbergh D (2014). Long-range orientation and atomic attachment of nanocrystals in 2D honeycomb superlattices. Science.

[4] Weidman MC, Smilgies DM, Tisdale WA (2016). Kinetics of the self-assembly of nanocrystal superlattices measured by real-time in situ X-ray scattering. Nat Mater.

[5] Abelson A, Qian C, Salk T, Luan Z, Fu K, Zheng J-G, Wardini JL, Law M (2020). Collective topo-epitaxy in the self-assembly of a 3D quantum dot superlattice. Nat Mater.

[6] Fan Z, Grünwald M (2019). Orientational order in self-assembled nanocrystal superlattices. J Am Chem Soc.

[7] Bentzon MD, van Wonterghem J, Mørup S, Thölén A, Koch CJW (1989). Ordered aggregates of ultrafine iron oxide particles: ‘super crystals’. Philos Mag B.

[8] Pileni MP (2007). Self-assembly of inorganic nanocrystals: fabrication and collective intrinsic properties. Accounts Chem Res.

[9] Zhong Z, Gates B, Xia Y, Qin D (2000). Soft lithographic approach to the fabrication of highly ordered 2D arrays of magnetic nanoparticles on the surfaces of silicon substrates. Langmuir.

[10] Ibanez M, Luo Z, Genc A, Piveteau L, Ortega S, Cadavid D, Dobrozhan O, Liu Y, Nachtegaal M, Zebarjadi M, Arbiol J, Kovalenko MV, Cabot A (2016). High-performance thermoelectric nanocomposites from nanocrystal building blocks. Nat Commun.

[11] Bockstaller MR, Kolb R, Thomas EL (2001). Metallodielectric photonic crystals based on diblock copolymers. Adv Mater.

[12] Courty A, Mermet A, Albouy P, Duval E, Pileni MP (2005). Vibrational coherence of self-organized silver nanocrystals in f.c.c. supra-crystals. Nat Mater.

[13] Kang Y, Ye X, Chen J, Cai Y, Diaz RE, Adzic RR, Stach EA, Murray CB (2013). Design of Pt–Pd binary superlattices exploiting shape effects and synergistic effects for oxygen reduction reactions. J Am Chem Soc.

[14] Kagan CR, Lifshitz E, Sargent EH, Talapin DV (2016). Building devices from colloidal quantum dots. Science.

[15] Whitham K, Yang J, Savitzky BH, Kourkoutis LF, Wise F, Hanrath T (2016). Charge transport and localization in atomically coherent quantum dot solids. Nat Mater.

[16] Schulz F, Pavelka O, Lehmkühler F, Westermeier F, Okamura Y, Mueller NS, Reich S, Lange H (2020). Structural order in plasmonic superlattices. Nat Comm.

[17] Motte L, Billoudet F, Pileni MP (1995). Self-assembled monolayer of nanosized particles differing by their sizes. J Phys Chem.

[18] Weller H (1996). Self-organized superlattices of nanoparticles. Angew Chem.

[19] Whetten RL, Khoury JT, Alvarez MM, Murthy S, Vezmar I, Wang ZL, Stephens PW, Cleveland CL, Luedtke WD, Landman U (1996). Nanocrystal gold molecules. Adv Mater.

[20] Haubold D, Reichhelm A, Weiz A, Borchardt L, Ziegler C, Bahrig L, Kaskel S, Ruck M, Eychmüller A (2016). The formation and morphology of nanoparticle supracrystals. Adv Funct Mater.

[21] Mirkin CA, Letsinger RL, Mucic RC, Storhoff JJ (1996). A DNA-based method for rationally assembling nanoparticles into macroscopic materials. Nature.

[22] Macfarlane RJ, Lee B, Jones MR, Harris N, Schatz GC, Mirkin CA (2011). Nanoparticle superlattice engineering with DNA. Science.

[23] Daniel MB, Tyler A, Dunbar DMS, Tobias H (2020). Coupled dynamics of colloidal nanoparticle spreading and self-assembly at a fluid–fluid interface. Langmuir.

[24] Lin H, Song L, Huang Y, Cheng Q, Chen T (2020). Macroscopic Au@PANI core/shell nanoparticle superlattice monolayer film with dual-responsive plasmonic switches. ACS Appl Mat Inter.

[25] Partridge JG, Scott S, Dunbar ADF, Schulze M, Brown SA, Wurl A, Blaikie RJ (2004). Formation of electrically conducting mesoscale wires through self-assembly of atomic clusters. IEEE Trans Nanotechnol.

[26] Li D, Freitag M, Pearson J, Qiu ZQ, Bader SD (1994). Magnetic phases of ultrathin Fe grown on Cu(100) as epitaxial wedges. Phys Rev Lett.

[27] Teichert C (2002). Self-organization of nanostructures in semiconductor heteroepitaxy. Phy Rep.

[28] Carroll SJ, Palmer R, Mulheran P, Hobday S, Smith R (1998). Deposition and diffusion of size-selected (Ag_400_^+^) clusters on a stepped graphite surface. Appl Phys A.

[29] Shi Z, Han M, Song F, Zhou J, Wan J, Wang G (2006). Hierarchical self-assembly of silver nanocluster arrays on triblock copolymer templates. J Phys Chem B.

[30] Lopes W, Jaeger HM (2001). Hierarchical self-assembly of metal nanostructures on diblock copolymer scaffolds. Nature.

[31] Elafandi S, Ahmadi Z, Azam N, Mahjouri-Samani M (2020). Gas-phase formation of highly luminescent 2D GaSe nanoparticle ensembles in a nonequilibrium laser ablation process. Nanomaterials.

[32] Wegner K, Piseri P, Tafreshi HV, Milani P (2006). Cluster beam deposition: a tool for nanoscale science and technology. J Phys D Appl Phys.

[33] Binns CW (2001). Nanoclusters deposited on surfaces. Surf Sci Rep.

[34] Jensen P (1999). Growth of nanostructures by cluster deposition: experiments and simple models. Rev Mod Phys.

[35] Vajda S, White MG (2015). Catalysis applications of size-selected cluster deposition. ACS Catal.

[36] Bardotti L, Jensen P, Hoareau A, Treilleux M, Cabaud B (1995). Experimental observation of fast diffusion of large antimony clusters on graphite surfaces. Phys Rev Lett.

[37] Chen JB, Zhou JF, Häfele A, Yin CR, Kronmüller W, Han M, Haberland H (2005). Morphological studies of nanostructures from directed cluster beam deposition. Eur Phys J D.

[38] Brechignac C, Cahuzac P, Carlier F, Colliex C, Leroux J, Masson A, Yoon B, Landman U (2002). Instability driven fragmentation of nanoscale fractal islands. Phys Rev Lett.

[39] Han M, Xu C, Zhu D, Yang L, Zhang J, Chen Y, Ding K, Song F, Wang G (2007). Controllable synthesis of two-dimensional metal nanoparticle arrays with oriented size and number density gradients. Adv Mater.

[40] Klecha E, Ingert D, Pileni MP (2010). How the level of ordering of 2D nanocrystal superlattices is controlled by their deposition mode. J Phys Chem Lett.

[41] Gutiérrez-Wing C, Santiago P, Ascencio JA, Camacho A, José-Yacamán M (2000). Self-assembling of gold nanoparticles in one, two, and three dimensions. Appl Phys A Mater Sci Process.

[42] Yoon B, Akulin VM, Cahuzac P, Carlier F, de Frutos M, Masson A, Mory C, Colliex C, Bréchignac C (1999). Morphology control of the supported islands grown from soft-landed clusters. Surf Sci.

[43] He L, Chen X, Mu Y, Song F, Han M (2010). Two-dimensional gradient Ag nanoparticle assemblies: multiscale fabrication and SERS applications. Nanotechnology.

[44] Wang ZL (1998). Structural analysis of self-assembling nanocrystal superlattices. Adv Mater.

[45] Murray CB, Kagan CR, Bawendi MG (2000). Synthesis and characterization of monodisperse nanocrystals and close-packed nanocrystal assemblies. Annu Rev Mater Sci.

[46] Puntes VF (2001). Colloidal nanocrystal shape and size control: the case of cobalt. Science.

