# Supplementary material for: Formation Mechanism of Well-Ordered Densely Packed Nanoparticle Superlattices Deposited from Gas Phase on Template-Free Surfaces
Source: Nanoscale Res Lett. 2021 Nov 30;16:172. doi: 10.1186/s11671-021-03635-7 (PMC8633269; doi:10.1186/s11671-021-03635-7)
Supplement: Supplementary file 1 — Additional file 1. The following files are available free of charge. Size distribution of Fe nanoparticles; the element distribution of Fe nanoparticle arrays; oxidation state of Fe nanoparticle arrays; heavily deposited Fe nanoparticle film; Fe nanoparticle arrays on the surface with different defect densities; surface morphology of amorphous carbon films; Fe nanoparticle aggregates deposited under nonoptimum condition; Note 1. On the rigid hard structure formed with metal nanoparticles without surfactant; Note 2. On the effects of surface texture of the amorphous carbon substrate. [file 11671_2021_3635_MOESM1_ESM.doc]

Additional file

Formation Mechanism of Well-ordered Densely Packed Nanoparticle Superlattices Deposited from Gas Phase on Template-free Surfaces

Chang Liu^1,2^, Fei Liu^1,2^, Chen Jin^1,2^, Sishi Zhang^2^, Lianhua Zhang^1^, Min Han*^1,2^

*^1^National Laboratory of Solid State Microstructures and Collaborative Innovation Centre of Advanced Microstructures, Nanjing University, Nanjing 210093, China*

*^2^Department of Materials Science and Engineering and Jiangsu Key Laboratory of Artificial Functional Materials, Nanjing University, Nanjing 210093, China*

**Correponding author*

*E-mail address: sjhanmin@nju.edu.cn.*

**Size distribution of Fe nanoparticles**

The size distribution of the deposited nanoparticles is determined from the TEM image based on a statistical analysis using a minimum of 300 nanoparticles. Histograms of the sizes of the Fe nanoparticles in the samples prepared with different deposition rates are shown in Additional file 1: Fig. S1. The size distribution can be approximatively fitted with a log-normal function.


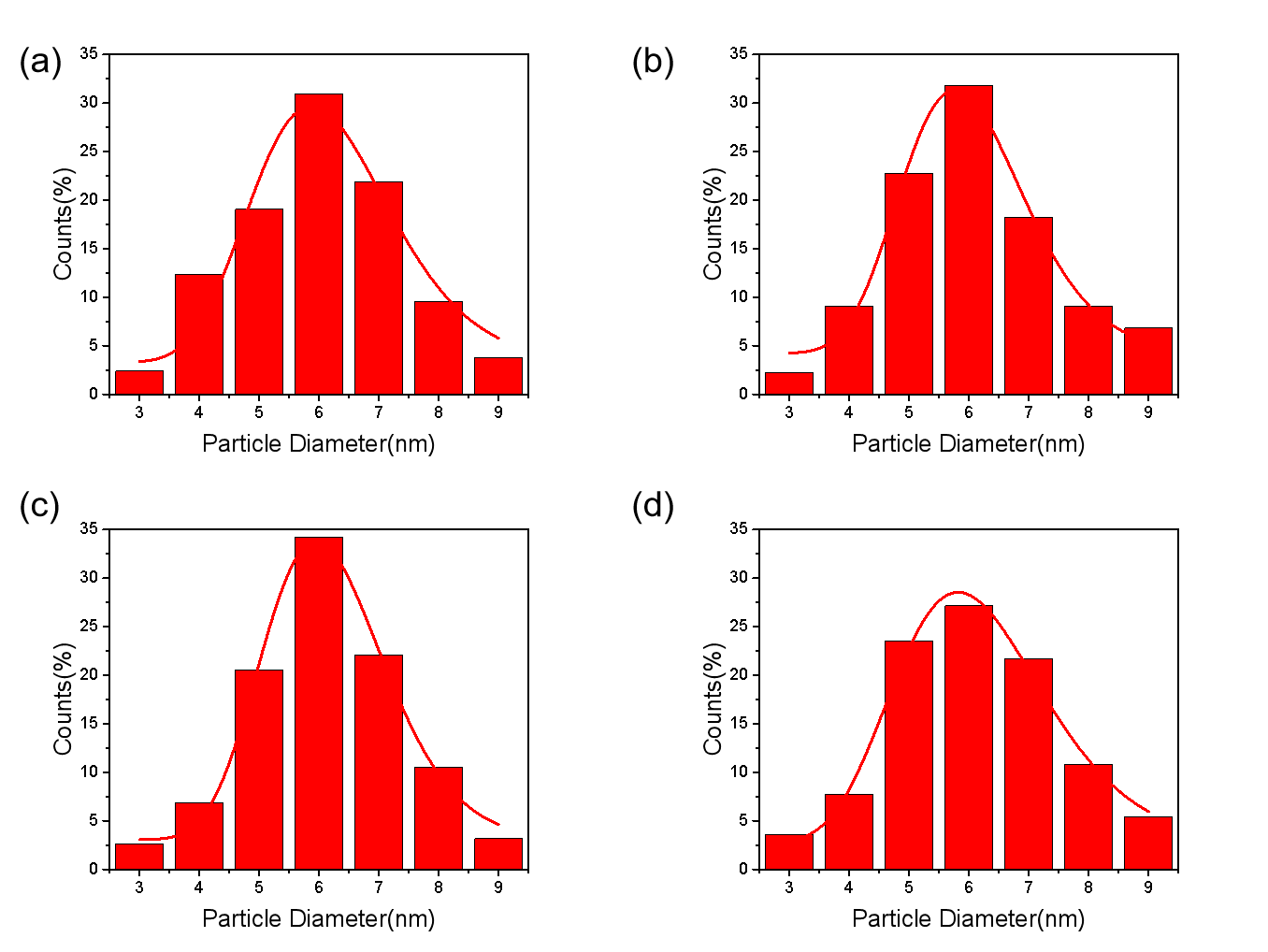


**Additional file 1: Fig. S1.** Size distributions of Fe nanoparticles in the samples prepared with different deposition rates (a) 0.1Å∙s^−1^ (b) 0.3Å∙s^−1^ (c) 0.5Å∙s^−1^(d) 0.7Å∙s^−1^.

**The element distribution of Fe nanoparticle arrays**

The Fe nanoparticle arrays are analyzed using Energy Dispersive X-ray spectroscopy (EDX) to confirm the composition of the nanoparticles. Additional file 1: Fig. S2a shows a typical High-Angle Annular Dark-Field (HAADF) image of the ordered Fe nanoparticle arrays, on which elemental mapping analysis is carried. Additional file 1: Fig. S2b,c show the corresponding EDX elemental mappings of Fe and O, respectively. It is clear that O always presents accompanying with Fe, revealing that the nanoparticles are oxidized. The merged image (Additional file 1: Fig. S2d) further approves the mainly overlapping of the two elements.


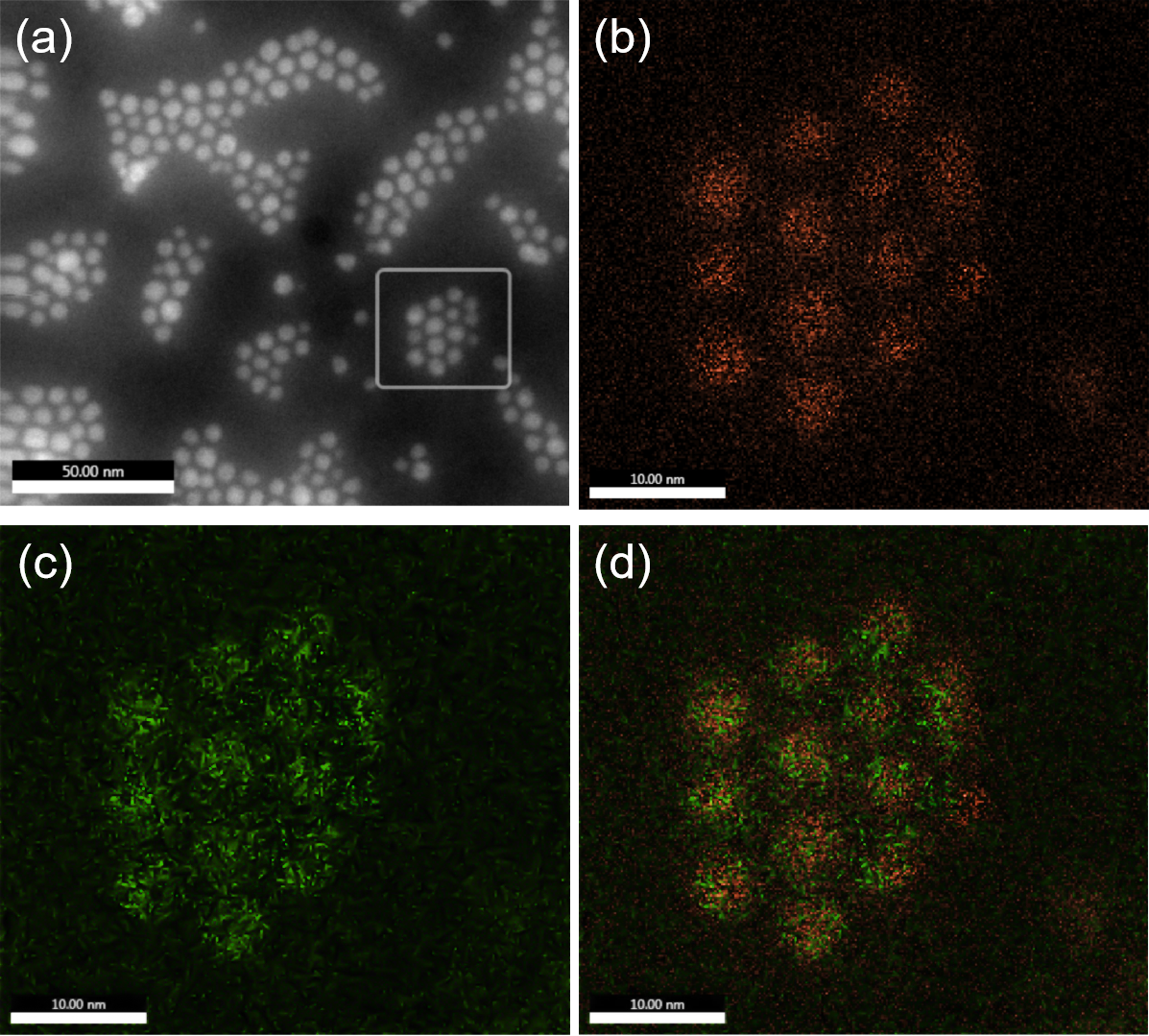


**Additional file 1: Fig. S2.** HAADF image (a) of the Fe nanoparticle arrays and EDX elemental mapping images of the marked area for Fe element (b) and O element (c) as well as the merge of Fe and O (d).

**Oxidation state of Fe nanoparticle arrays**

To analyze the oxidation state of the Fe nanoparticle arrays, X-ray Photoelectron Spectroscopy (XPS) was carried out with an ESCALABMK-II spectrometer using a monochromatic Mg Kα source. Additional file 1: Fig. S3 shows the photoemission data of the Fe 2p core levels measured from the Fe nanoparticle arrays before and after Ar^+^ sputtering. Before Ar^+^ sputtering, the peaks of Fe 2p3/2 and Fe 2p1/2 core levels are mainly contributed by Fe_2_O_3_ (with a binding energy of 710.2eV and 723.6eV, respectively). Only very weak peaks related to pure Fe (with a binding energy of 706.3eV for 2p3/2 core level and 719.6eV for 2p1/2 core level) can be decomposed. After Ar^+^ sputtering, which removes a certain number of atom layers from the nanoparticle surface, photoemissions from pure Fe become the main contributors of the XPS peaks instead, as can be seen from Additional file 1: Fig. S3. The result clearly demonstrates the Fe core and Fe oxide shell structure of the nanoparticles.





**Additional file 1: Fig. S3.** XPS Fe 2p core level spectrum of the Fe nanoparticle assembled arrays before and after Ar^+^ cleaning.

**Heavily deposited Fe nanoparticle film**

To indicate to the influence of coverage on nanoparticle array order, a heavily deposited Fe nanoparticle film performed with a deposition rate of 0.7Å∙s^−1^ (the same of Additional file 1: Fig.3c) is shown in Additional file 1: Fig. S4. The increase of deposition mass can not improve the order of nanoparticle array at high deposition rate.


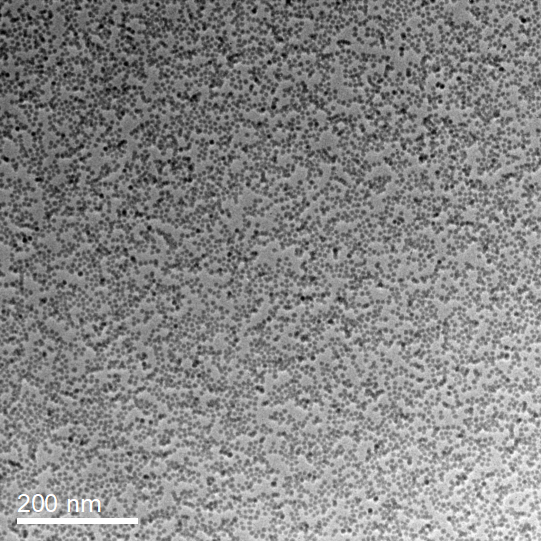


**Additional file 1: Fig. S4.** TEM image of Fe nanoparticle arrays deposited on a Formvar film with a deposition rate of 0.7Å∙s^−1^.

**Fe nanoparticle arrays on the surface with different defect density**

We obtained Fe nanoparticle arrays as shown in the Additional file 1: Fig. S5 on the surface of carbon films with different defect densities. With the deposition rate 0.15Å∙s^−1^, the scale of the ordered densely-packed nanoparticle domains is significantly smaller at higher surface defect density in the upper left region with higher defect density, while the scale of domains in the lower right is larger.

**Additional file 1: Fig. S5.** TEM image of Fe nanoparticle arrays deposited on an amorphous carbon film with a deposition rate of 0.15Å∙s^−1^.

**Surface morphology of amorphous carbon films**

In order to determine the defect features on amorphous carbon film surface, Atomic Force Microscope (AFM) were used to characterize the surface morphology of the substrate. The AFM topographic image in Figure shows that there are no ordered arrays of nano-scale defects on the amorphous carbon films.


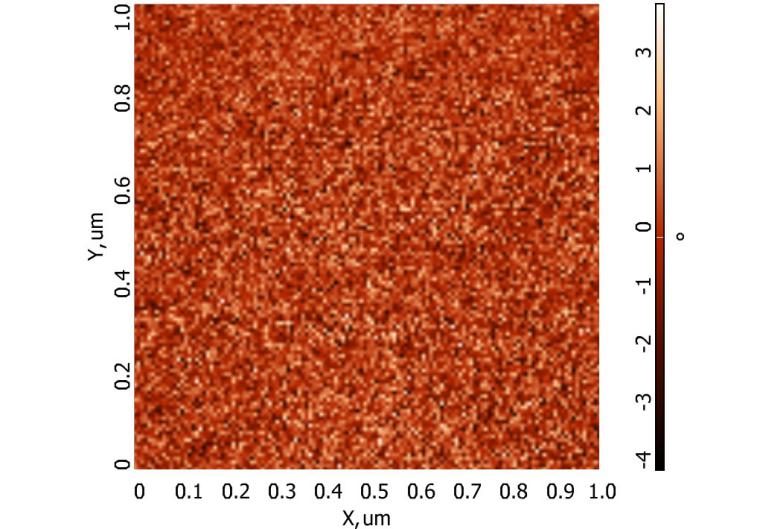


**Additional file 1: Fig. S6.** AFM image of amorphous carbon film surface.

In fact, if there exists surface roughness which is compatable to the nanoparticle size on scale, it is easy to be distinguished from the TEM or AFM images. As shown in Additional file 1: Fig. S7a and Additional file 1: Fig. S7b, those nanometer-sized amorphous carbon particles deposited on the amorphous carbon film is clearly visible in the TEM or AFM images.


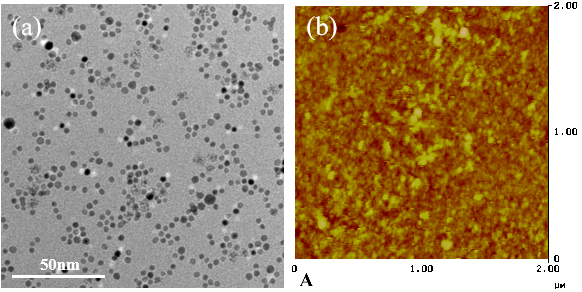


**Additional file 1: Fig. S7.**  (a) TEM and (b) AFM image of amorphous carbon nanoparticles deposited on amorphous carbon film surface.

**Fe nanoparticle aggregates deposited under non-optimum condition**

When non-optimum deposition conditions is employed, non-ordered aggregates of nanoparticles are formed, In Additional file 1: Fig. Sc, a TEM image of such a Fe nanoparticle film is shown. The film is prepared with a deposition rate of 0.5Å∙s^−1^.

**Additional file 1: Fig. S8.** TEM image of Fe nanoparticle arrays deposited on an amorphous carbon film with a deposition rate of 0.5Å∙s^−1^.

**Additional file 1: Note 1: On the rigid hard structure formed with metal nanoparticles without surfactant**

When the surfaces of metal nanoparticles are not encapsulated with surfactant molecules, the interactions between the closely-spaced nanoparticles are mainly attractive van der Waals forces, which drive the self-assembling of the densely-packed nanoparticle arrays. From TEM image, the diameter (D) of Fe nanoparticles is about 6nm, and the space (a) between the neighbouring nanoparticles is on the order of 0.1nm. The van der Waals attractive energy ϕ of the neighbouring nanoparticles can be calculated as [1]: ϕ ~AD/(24a), where A is Hamakar constant. For metal nanoparticles, the Hamakar constant is about 3×10^-19^J [2]. Therefore a van der Waals attractive energy as high as ~8eV can be calculated. Taking into account the fact that the neighbouring nanoparticles are closely contacted in the lattices and the attractive energy is relatively high, we state that a rigid hard structure is formed. On the other hand, in the superlattices assembled from thiol-passivated nanoparticles, the distance between metal-passivated particles is determined by the length of the thiolate molecule, which is typically 1-2nm. This dimension is obviously larger than the range over which the attraction forces between metal nanoparticles are active. Therefore the main contribution to the interaction in the superlattice comes from the thiolate molecules rather than from the metal nanoparticle cores. The van der Waals interaction between thiol molecules dominates the crystallization process and the interparticle distance. According to molecular dynamics simulation[3], the cohesion energy of passivated nanoparticle superlattices (the difference between the total energy of the isolated nanoparticle and its energy in the superlattice) can reach ~4eV, while the relative contribution of the nanoparticle-nanoparticle van der Waals interaction to the intercrystallite potential energy is ~1%. In the sense that the presence of the surfactant molecules results in a steric repulsion between nanoparticles and a lower NP-NP affinity, the structure of the superlattice assembled from thiol-passivated nanoparticles is relatively soft, as has been demonstrated experimentally [4].

**Additional file 1: Note 2: On the effects of surface texture of the amorphous carbon substrate**

The texture of the carbon substrate will significantly affects the deposition pattern of the nanoparticle monolayers. For example, the concave bends in graphite surfaces can act as linear trappings of nanoparticles and guide them to a quasi-one dimensional particle gas [5]. On the surface of highly oriented pyrolytic graphite (HOPG), metal nanoparticles usually decorate step edges to form chain-like morphologies if the surface defect density is low, or be trapped on point defects on the terraces and form fractal-like structures or even random distributed arrays, with the increase of the surface defect densities. Similar, the texture of the amorphous carbon substrate can also affect the aggregation morphology of the deposited nanoparticles. If the amorphous carbon substrate contains high density of point defects, which can be generated by ion irradiation or even keV energetic nanoparticle impactions [6], the nanoparticles are mostly pinned on the surface defect sites so that the morphology of the deposits has the feature of random deposition. In our work, only randomly distributed nanoparticle arrays can be obtained on the Formvar film surface without any treatment (Fig. 4). This means there is no texture on the Formvar film surface. Since the amorphous carbon substrates we used are deposited on the Formvar films, we can believe that the formation of the densely-packed ordered monolayer structure on the amorphous carbon substrate is not resulted from a decoration of the amorphous carbon substrate, since such texture cannot be formed on the amorphous carbon film grow from texture-free Formvar support. In the TEM image shown in Additional file 1: Fig. S5, the effect of surface defect density on the morphologies of the nanoparticle aggregates can be clearly seen. Under the same deposition condition, the scale of the ordered densely-packed nanoparticle domains is significantly smaller at higher surface defect density. We therefore conclude that a smooth surface is most conducive to improve the order of the array.

**References**
1. Bansal SA, Kumar V, Karimi J, Singh AP, Kumar S (2020) Role of gold nanoparticles in advanced biomedical applications. Nanoscale Adv 2: 3764–3787.

2. Jiang K, Pinchuk P(2016) Temperature and size-dependent Hamaker constants for metal nanoparticles. Nanotechnology 27:345710.

3. Luedtke WD, Landman U (1996) Structure, Dynamics, and Thermodynamics of Passivated Gold Nanocrystallites and Their Assemblies. J Phys Chem 100: 13323-13329.

4. Gutiérrez-Wing C, Santiago P, Ascencio JA, Camacho A,José-Yacamán M (2000) Self-assembling of gold nanoparticles in one, two, and three dimensions. Appl Phys A Mater Sci Process 71: 237-243

5. Schmidt M, Kebaieli N, Lando A, Benrezzak S, Baraton L, Cahuzac, Masson A,Brechignac C (2008) Bent graphite surfaces as guides for cluster diffusion and anisotropic growth. Phys Rev B 77: 205420.

6. Chen JB, Zhou JF, Häfele A, Yin CR, Kronmüller W, Han M,Haberland H (2005) Morphological studies of nanostructures from directed cluster beam deposition. Eur Phys J D 34: 251-254.
